# MBRA-2: a Modified Chemostat System to Culture Biofilms

**DOI:** 10.1128/spectrum.02928-22

**Published:** 2022-12-07

**Authors:** Justin N. Jens, Daniel J. Breiner, Rachel L. Neve, Matilda M. Fiebig, Vanessa V. Phelan

**Affiliations:** a Department of Pharmaceutical Sciences, Skaggs School of Pharmacy and Pharmaceutical Sciences, University of Colorado Anschutz Medical Campus, Aurora, Colorado, USA; b Department of Immunology and Microbiology, School of Medicine, University of Colorado Anschutz Medical Campus, Aurora, Colorado, USA; University of Nebraska-Lincoln

**Keywords:** MBRA-2, *Pseudomonas aeruginosa*, biofilms, chemostat cultures

## Abstract

Culture-dependent approaches for investigating microbial ecology aim to model the nutrient content of specific environments by simplifying the system for high-resolution molecular analysis. These *in vitro* systems are enticing due to their increased throughput compared to animal models, flexibility in modulating nutrient content and community composition, scaling of culture volume to isolate biological molecules, and control of environmental parameters, such as temperature, humidity, and nutrient flow. However, different devices are used to investigate homogenous, planktonic microbial communities and heterogeneous biofilms. Here, we present the minibioreactor array 2 (MBRA-2) with media rails, a benchtop multireactor system derived from the MBRA system that enables researchers to use the same system to grow planktonic and biofilm cultures. We simplified flow through the system and reduced contamination, leakage, and time required for array assembly by designing and implementing a reusable media rail to replace the branched tubing traditionally used to convey media through chemostat arrays. Additionally, we altered the structure of the six-bioreactor strip to incorporate a removable lid to provide easy access to the bioreactor wells, enabling biofilm recovery and thorough cleaning for reuse. Using Pseudomonas aeruginosa, a model biofilm-producing organism, we show that the technical improvements of the MBRA-2 for biofilms growth does not disrupt the function of the bioreactor array.

**IMPORTANCE** The MBRA-2 with media rails provides an accessible system for investigators to culture heterogenous, suspended biofilms under constant flow.

## INTRODUCTION

Biofilms are diverse, sessile microbial communities that grow embedded in a self-produced matrix (1). Biofilms persist in many environments within the human body and often lead to the development of chronic infections, which are recalcitrant to antimicrobial treatment and the host immune response (2). These medically relevant biofilms exist in two forms: surface-attached biofilms, such as dental plaque, or micro-aggregate biofilms, like those measured from the sputum of persons with cystic fibrosis (CF) (3).

To model biofilm formation *in vitro*, several systems have been developed (3–7), including closed models, such as on agar (8, 9) or in well plates under nutrient- and aeration-limited conditions (10), and open models with continuous nutrient flow, including plug flow reactors (8, 9, 11, 12), constant depth film fermenters (13), drip-flow biofilm reactors (14), rotating disk reactors (15), CDC biofilm reactors (16), microfluidic devices (17–20), and the modified Robbins device (21). While these platforms have been widely applied to investigating biofilm growth on the surface of well plates or coupons submerged in culture medium, it is challenging to grow microaggregate biofilms under these conditions.

Recently, several variations of intermediate-size chemostats have been designed and implemented to study microbial ecology (22–28). Intermediate-size microbial bioreactors are scaled-down versions of stirred-tank reactors that support culture volumes ranging from 5 to 50 mL and are cheaper and easier to implement than standard chemostats (volumes of 0.25 to 10 L) (29, 30). Previous studies have shown that compromising the steady-state equilibrium of chemostats through suspension or immersion of coupons in the medium disrupts culture homogeneity and enables biofilm to increase overtime (31, 32). Therefore, the intention of this study was to modify an existing intermediate-size chemostat to be compatible with culturing biofilms.

Herein, we present the MBRA-2, a continuous-culture system derived from the minibioreactor array (MBRA) system. Although there are many variations of intermediate-size bioreactors, we decided to modify the MBRA system to support biofilm growth due to the ease of fabrication and assembly, ability to reuse the reactor strips, and small footprint of the assembled array (28, 33, 34). Using Pseudomonas aeruginosa as a model biofilm-forming organism (35), we altered the design of the MBRA to better support biofilm growth and recovery. We simplified flow through the system by designing and implementing a reusable media rail to replace the branched tubing used to convey media through the system. The media rails can be used with any chemostat array and reduce contamination, leakage, and time required for array assembly. Additionally, we designed the MBRA-2 reactor strip to incorporate a removable lid to provide easy access to the bioreactor wells, enabling biofilm recovery and thorough cleaning for reuse. The MBRA-2 with media rails is easily assembled and provides an opportunity to grow microbes as either homogeneous cultures when stirred or as biofilms under nonmixing conditions. Importantly, the technical improvements of the MBRA-2 to support biofilm growth did not disrupt the function of the chemostat array, as evidenced by the ability to grow P. aeruginosa at similar levels to the original bioreactor array.

## RESULTS

### Evaluation of MBRA for aerobic P. aeruginosa cultures.

**FIG 1:**
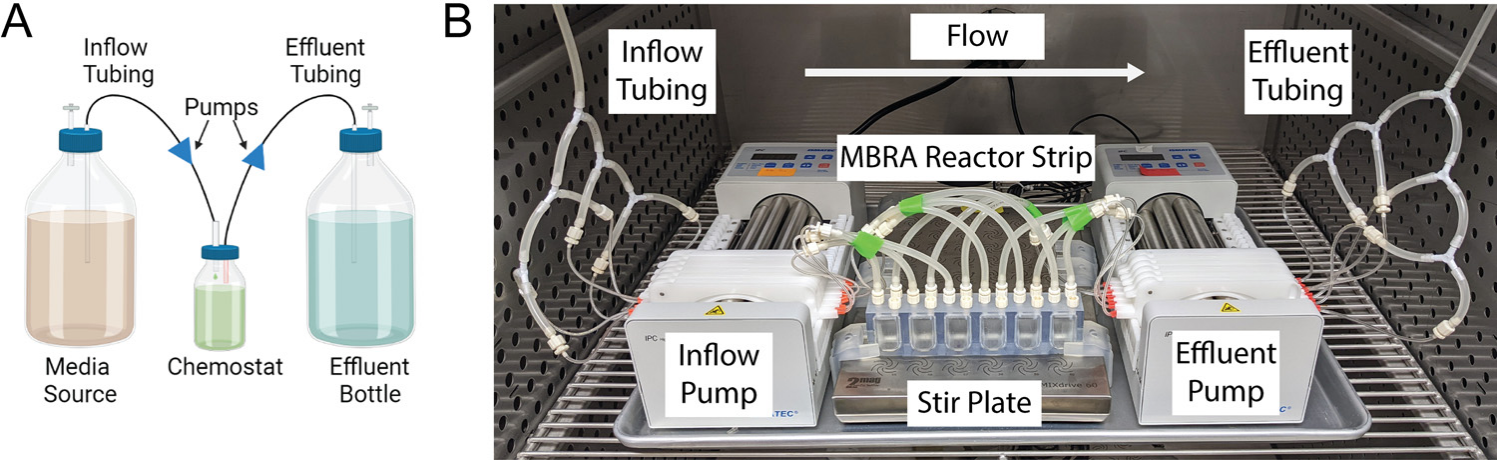
(A) Chemostats are stirred-tank bioreactors with continuous nutrient flow. (B) A minibioreactor array (MBRA) reactor strip consists of six 25-mL reactor wells. Each reactor well has three ports: an inflow port, an effluent port, and a sampling port. Up to four MBRA reactor strips (24 reactors) can be situated on a 60-spot magnetic stir plate. Media flow to and from the reactor wells is controlled by two 24-channel peristaltic pumps. Each reactor well is connected to the media source and the effluent bottle through a series of branched tubing.

To evaluate whether simple disruption of culture homogeneity would enable the biofilm growth by P. aeruginosa in the existing MBRA, reactor strips were fabricated, and the array was assembled following the detailed instructions (28). An MBRA reactor strip comprised of six 25-mL reactor wells was fabricated using thermoset polymer as a single unit (Fig. 1). The reactor strip was situated on a 60-spot magnetic stir plate using 3D-printed holders and flow through the MBRA reactor wells was maintained by multichannel peristaltic pumps. Sterile media was introduced by branched tubing to the growing cultures through the inflow port and waste was removed through the effluent port.

**FIG 2:**
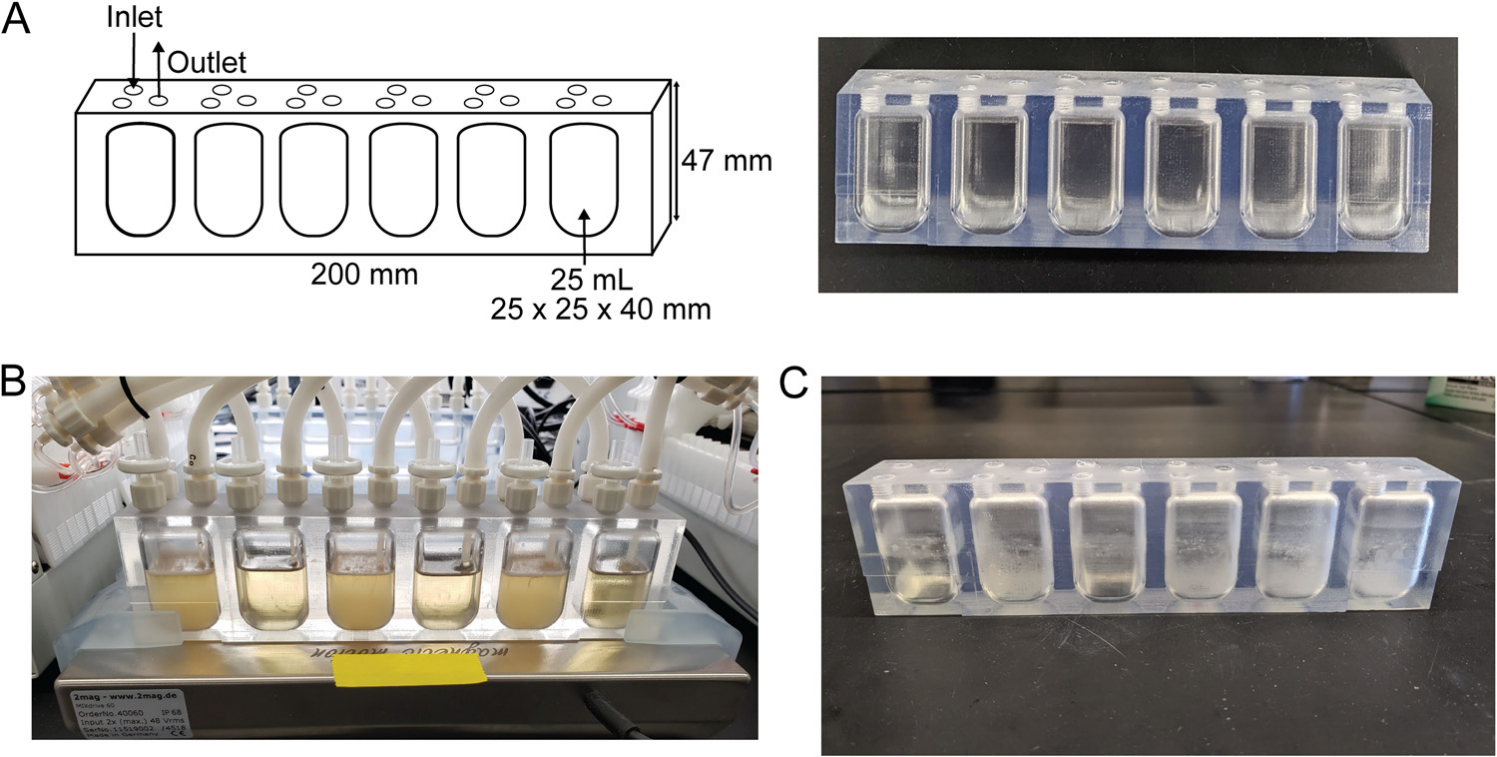
(A) MBRA reactor strip design, dimensions, and fabricated reactor strip. (B) P. aeruginosa PAO1 biofilm growth in MBRA (reactor wells 1, 3, and 5). (C) Biofilm residue in an MBRA reactor strip after cleaning following reported methods.

Prior to inoculation, sterile Luria Broth (LB) was introduced into the MBRA, and the sterility of the system was validated. The media was inoculated with P. aeruginosa PAO1 through the sampling port and a biofilm was allowed to grow under low flow, nonstirring conditions at 37°C for 3 days. P. aeruginosa PAO1 grew robustly (Fig. 2A), with a clearly distinguishable biofilm at the air-liquid interface. The biofilm strongly adhered to the internal edges of the MBRA reactor wells, preventing recovery of the biofilm through the sampling port (Fig. 2B). To clean the MBRA reactor strip, the previously described cleaning protocol was followed (27). However, due to limited accessibility of the reactor wells for manual cleaning, it was not possible to remove the residual biofilm from the reactor strip (Fig. 2C). Therefore, to enable use and reuse of the MBRA reactor strips for biofilm growth, it was necessary to alter its design by incorporating a removable lid.

### MBRA-2 reactor strip design, fabrication, and assembly.

**FIG 3:**
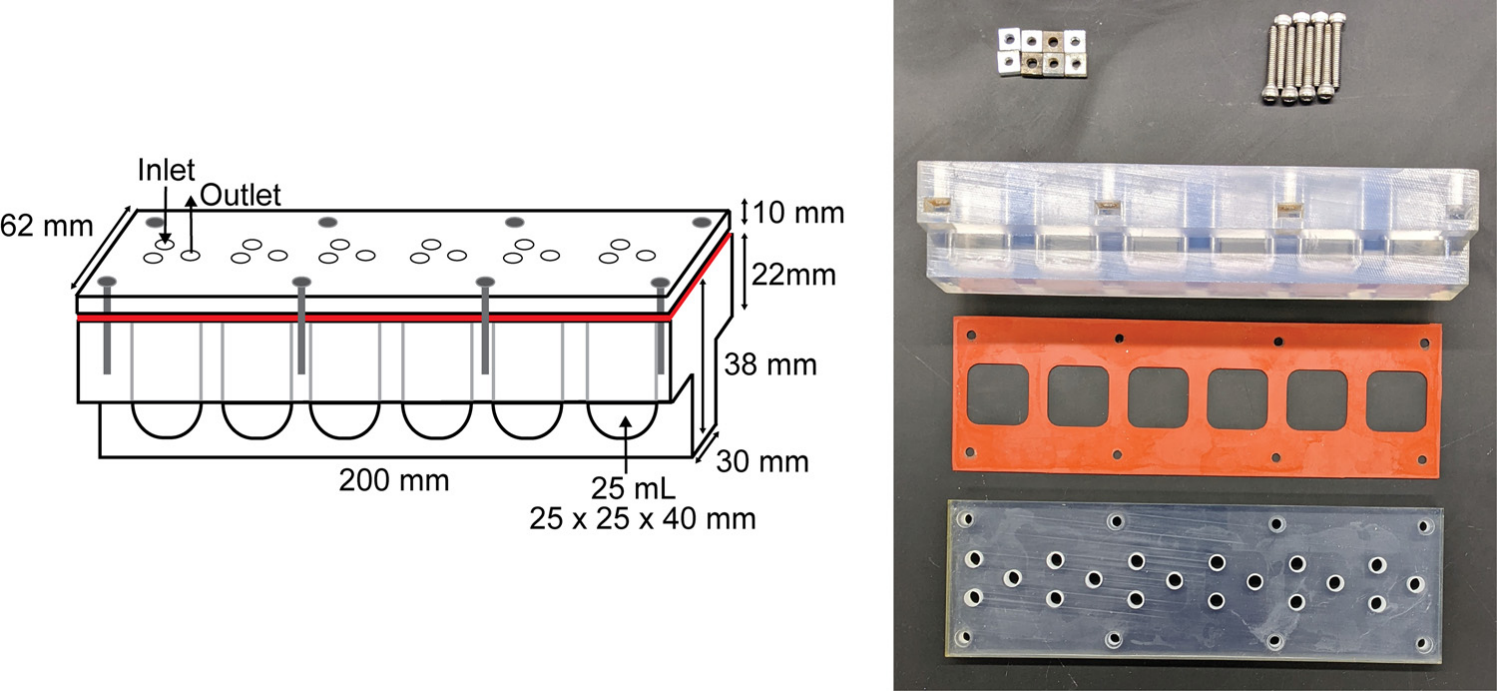
MBRA-2 reactor strip design, dimensions, and fabricated reactor strip components (reactor strip, gasket, lid, screws, and square nuts).

To incorporate a lid into the design of the reactor strip, we took into consideration the simplicity of the original design and our desire to retain its general features, including three ports for flow and sampling, volume of the reactor wells, size of the assembled MBRA, and compatibility with the holders that anchor the reactor strip to the stir plate. To maintain these features, the MBRA-2 reactor strip was designed as three components: a 3D printed 38-mm tall reactor strip consisting of six wells fabricated from thermoset polymer, a 3D printed 10-mm tall lid with three ports for each of the reactor wells also fabricated from thermoset polymer, and a 1-mm thick die-cast silicone gasket (Fig. 3).

To preserve the volume of the reactor wells and ensure the structural integrity of the MBRA-2 reactor strip, the width of the lid and upper 22 mm of reactor strip was increased from 30 mm to 62 mm. This design provided sufficient space to secure the silicone gasket between the lid and reactor strip using eight evenly spaced screws and square nuts, while also retaining the overall footprint of a fully assembled chemostat array. The width of the lower 16 mm of the reactor strip was maintained at 30 mm to provide compatibility of the assembled reactor strips with the holders that anchor the reactor strip to the stir plate. Importantly, as the height of the MBRA-2 reactor strip was conserved at 48 mm, the MBRA-2 reactor well dimensions (25 × 25 × 40 mm) and volume (25 mL) are identical to the MBRA reactor wells.

### Media rail design and fabrication.

**FIG 4:**
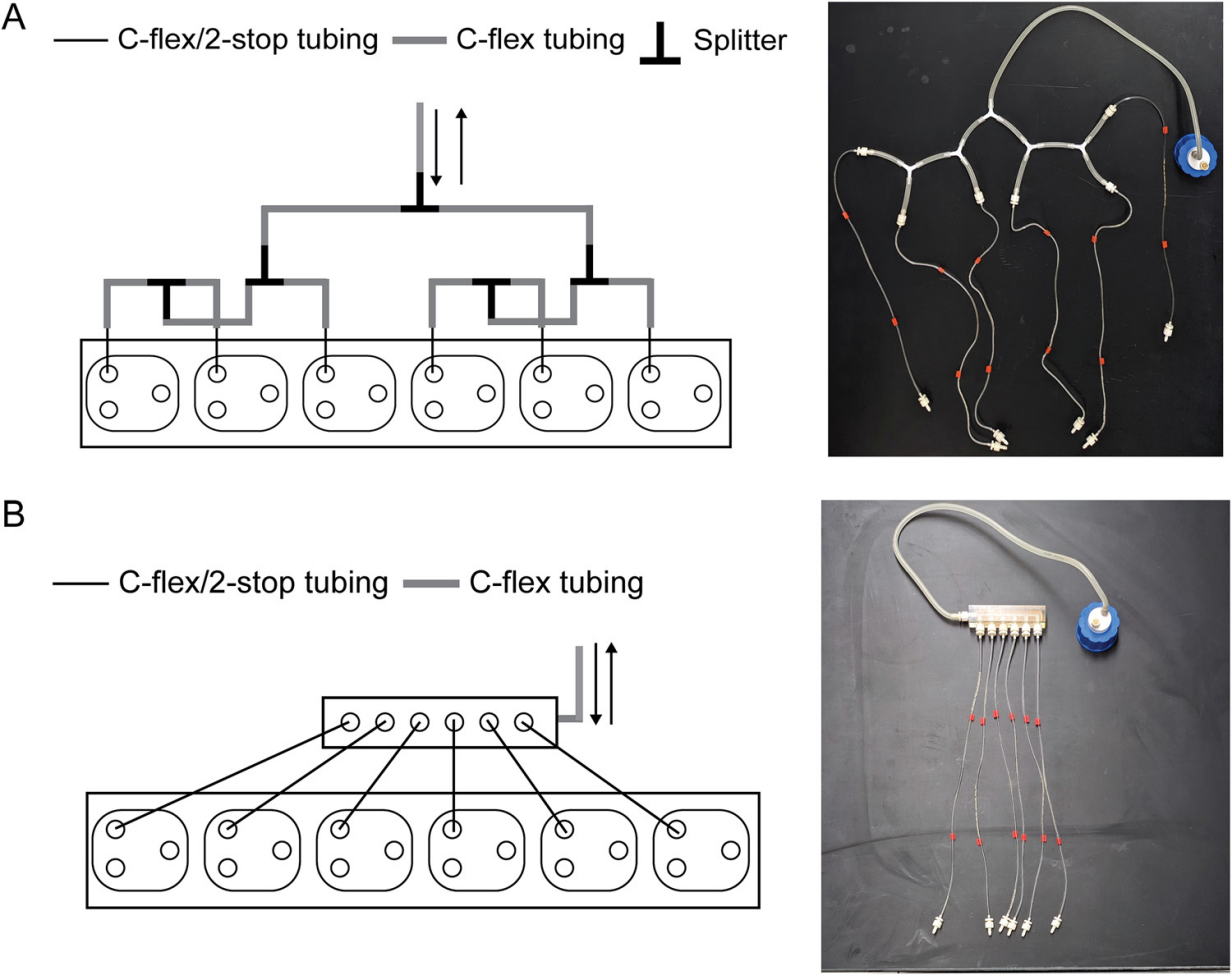
(A) Illustration and example of branched tubing. (B) Illustration and example of a media rail.

For most chemostat arrays, including the MBRA-2, multichannel peristaltic pumps are used to siphon media from a shared bottle to distribute it to multiple reactor wells. In a traditional array setup, media flow is supplied to multiple reactor wells from the media bottle by branched tubing, which divides the flow by splitting it from a single piece of tubing into two pieces of tubing using T- or Y- splitters and so on, until enough branches are created to provide media to all reactor wells (Fig. 4A). Since each chemostat array experiment requires a significant investment of time and resources, we aimed to minimize experimental failure due to the use of branched tubing to convey media flow, as each connection represented a potential point of contamination or flow disruption.

To simplify connecting multiple reactor wells to the same or multiple nutrient sources, we designed media rails (Fig. 4B). Reminiscent of automotive fuel rails that provide consistent gasoline flow from the fuel pump to multiple fuel injectors, media rails provide consistent flow from the media bottle to multiple reactor wells. Importantly, the media rail design is purposefully agnostic to flow directionality, enabling them to be used to either introduce media to or remove effluent from the bioreactor wells. Additionally, because the media rails are 3D printed using thermoset plastic, they can be reused after sterilization.

**FIG 5:**
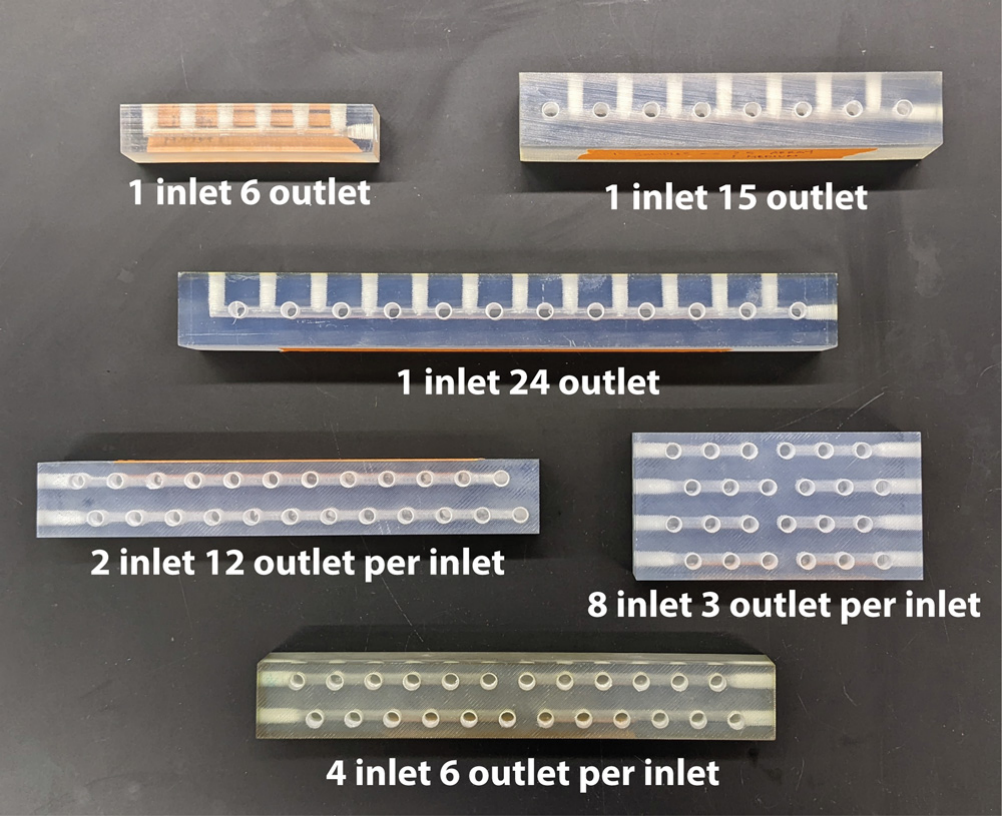
Representative examples of media rail configurations to support different experimental goals.

Although simplistic in design, the media rails provide a straightforward method for flexibility in experimental design using chemostat arrays (Fig. 5). Each media rail has at least one inlet port and six outlet ports to provide consistent flow from the media bottle to up to six bioreactor wells. However, media rails can be fabricated to meet the needs of an individual experiment. For example, a media rail designed to have one inlet port and 24 outlet ports provides the same nutrient conditions to 24 cultures. Alternatively, an experimental design consisting of replicate cultures (e.g., *n* = 3) cultured under eight different nutrient conditions is supported by a media rail configured with eight inlet ports and three outlet ports per inlet port. If fewer outlet ports are needed than the readily available media rails, the unused ports can simply be plugged. By replacing the branched tubing with media rails for chemostat array experiments, the time required for experimental setup is decreased, likelihood of experimental failure due to poor connections between tubing and splitters is diminished, and amount of plastic tubing waste generated is significantly reduced.

### Validation of MBRA-2 operation with media rails using P. aeruginosa.

**FIG 6:**
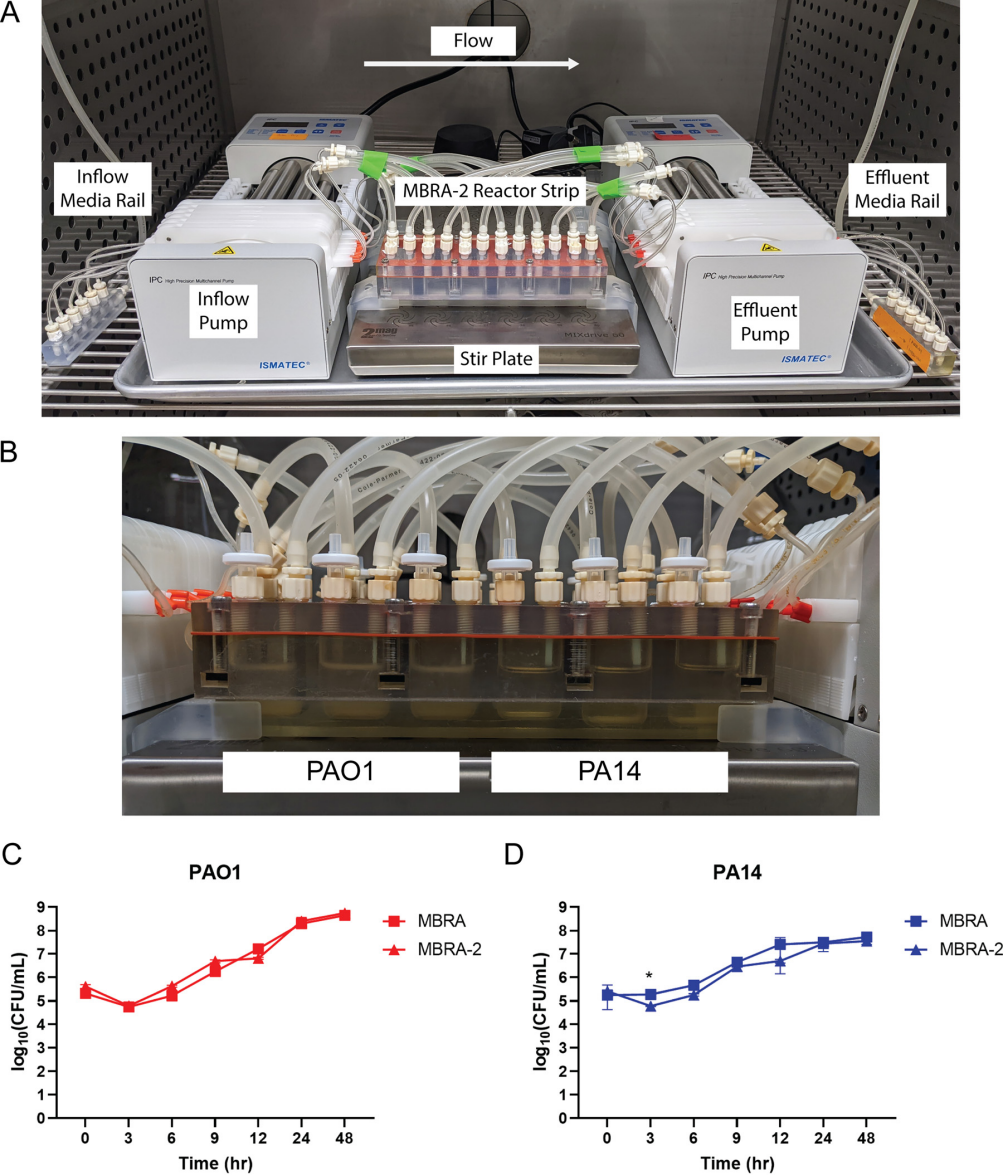
(A) Configuration of MBRA-2 with media rails. (B) Cultures of P. aeruginosa PAO1 (reactor wells 1 to 3) and PA14 (reactor wells 4 to 6) in an MBRA-2 reactor strip. (C) Enumerated growth of PAO1 (red, *n* = 3) and (D) PA14 (blue, *n* = 3) over 48 h growth in an MBRA (squares) and MBRA-2 (triangles). Statistical comparison of P. aeruginosa growth between MBRA and MBRA-2 was conducted using ANOVA with Šídák’s correction for multiple comparisons. Error bars indicate standard deviation. *, *P* < 0.05.

To compare growth of P. aeruginosa cultured in an MBRA-2 (MBRA-2 reactor strips and media rails, Fig. 6A) to the standard MBRA system (MBRA reactor strips and branched tubing, Fig. 1), both systems were assembled. A detailed description of the materials required as well as step by step directions for assembly of the MBRA-2 with media rails are available in supplemental material. The MBRA and MBRA-2 were operated in parallel under identical conditions. Prior to inoculation, sterile M9 minimal salts complemented with casamino acids (5 g/L) and 1 mM tryptophan was introduced into both systems and the sterility of the systems was validated. The modified M9 minimal salts media was used to ensure that P. aeruginosa could be accurately enumerated from the cultures. The media of three reactor wells for each system was inoculated in triplicate with P. aeruginosa PAO1 through the sampling port. Three additional reactor wells of each system were inoculated in triplicate with strain PA14. P. aeruginosa was allowed to grow under low flow, nonstirring conditions at 37°C for 2 days (Fig. 6B). Each reactor well was sampled at 3, 6, 9, 12, 24, and 48 h postinoculation and growth of PAO1 and PA14 was enumerated as CFU/mL (Fig. 6C and D).

Both strains of P. aeruginosa grew robustly in the chemostat array systems. While PA14 cultures were turbid with no clear formation of a biofilm, PAO1 cultures formed a biofilm at the air-liquid interface. After inoculation, both PAO1 and PA14 concentrations decreased by less than 1-log fold but recovered to inoculation levels within 6 h. There was no significant difference in PAO1 and PA14 growth between cultures in the MBRA and MBRA-2, except for a small, but statistically significant difference for PA14 at 3 h (*P* = 0.0424).

## DISCUSSION

The MBRA-2 reactor strip was designed to bridge chemostat and biofilm culture models. MBRA-2 reactor strips function as a continuous flow large volume well plate, which enables researchers to culture bacteria and fungi as homogenous, aerated planktonic communities when stirred or heterogeneous biofilm cultures when maintained under nonstirring conditions. The addition of a lid to the simply designed reactor strips allows for easier access to the reactor wells, permitting recovery of biofilms or microbial communities for further analysis as well as thorough cleaning. Although we fabricated the MBRA-2 reactor strips from thermostable plastic, repeated steam-based sterilization will eventually lead to cracking. However, the MBRA-2 reactor strip design is compatible with injection molding, which dramatically reduces the production cost of each reactor strip and enables fabrication of the reactor strips using longer lasting thermostable materials.

One of the limitations of standard chemostat arrays is the necessity to connect many reactor wells to a shared media source using branched tubing. Using branched tubing to support media flow through a fully assembled 24-reactor array requires 80 pieces of tubing and 40 splitters. Our media rails were designed to replace almost all this disposable tubing with a simple device capable of providing consistent flow to all reactor wells simultaneously. Importantly, different configurations of media rails can be designed to support individual experimental design needs and the media rails are agnostic to flow directionality, enabling them to be used for either media inflow or effluent outflow.

Like all bioreactor systems, including its predecessor, the design of the MBRA-2 has limitations. The MBRA-2 reactor wells only have three ports, two of which are used for nutrient flow, which limits the ability to integrate sensors into the system to monitor important environmental parameters, such as growth rate, dissolved oxygen, and pH. However, environmental conditions can be monitored using manual probes through the sampling port, from the outflow using a pH and CO_2_ sensor array (24), by adapting fluorescent sensors to the MBRA-2 system (22, 36), or by adding chemical indicators to the culture media (37). Further, the MBRA-2 relies on use of an environmental chamber or incubator sufficient in size to maintain constant atmosphere and temperature. Modifications to the system can be performed to allow the reactors to be maintained in a water bath (22, 27) or specially designed heat block (25) to maintain consistent temperature under atmospheric conditions. Lastly, although the lid of the MBRA-2 reactor strip provides access to the reactor wells, it is fabricated as a single piece of plastic, requiring the entire lid to be removed to access a specific reactor well. Widening the MBRA-2 reactor strip even further may provide enough space to incorporate individual lids for each reactor but may impact the ability to situate a fully assembled 24-reactor array on the stir plate.

The similar functionality of the MBRA-2 reactor strips and media rails to the original bioreactor array design was validated using P. aeruginosa strains PAO1 and PA14. Although this simple experiment highlights the ability to use the MBRA-2 system, further work will validate its ability to support communities. Nevertheless, addition of a lid to the reactor strip opens the possibility of other experimental designs in the MBRA-2 system that are not fully supported in traditional chemostats, including performing coupon-based biofilm studies (e.g., dental plaque on calcium hydroxyapatite disks) as these solid materials can now be recovered from the reactor wells. In this way, the MBRA-2 system supports the potential to further elucidate the complex relationship between microbes and their environment and the differences between planktonic and biofilm growth.

## MATERIALS AND METHODS

### Component fabrication.

MBRA-2 reactor strips, lids, and media rails were designed using 360 Fusion (Autodesk) computer-assisted design (CAD) software in consultation with ProtoLabs (www.protolabs.com). MBRA reactor strips and holders, MBRA-2 reactor strips and lids, and media rails were fabricated with DSM Somos Watershed 11122 resin by stereolithography by ProtoLabs from .stl files. The .stl files for the MBRA reactor strips and holders were kindly provided by Jennifer Auchtung (University of Nebraska). The silicone gasket was cut from silicone rubber sheeting (USA Sealing, Product ID 1MVT6) using a Cricut Maker 3 cutting machine fitted with a knife blade and a StrongGrip mat using the associated Design Space software. A thread-tapping tool was used to introduce 1/4″-28 threads into all ports, which were subsequently rinsed with water to remove residual resin dust. Fabrication files for the MBRA-2 reactor strips and lids, media rails, and gasket are available on Zenodo (10.5281/zenodo.6909500).

### MBRA and MBRA-2 assembly and operation.

The MBRA chemostat system was assembled as previously described (28). Detailed instructions and an instructional video for the assembly, sterilization, and operation of the MBRA-2 with media rails are in the supplemental material (Fig. S1 to S8, and Movie S1). Prior to use, the MBRA and MBRA-2 assemblies (including reactor strips, tubing, and media rails) were autoclaved. The MBRA and MBRA-2 reactor strips were situated on 60-spot stir plates (Millipore Sigma) and inflow and effluent two-stop pump tubing was locked into their respective peristaltic pumps. The sterile inflow media bottle cap was then screwed onto a bottle containing sterile media. Each MBRA and MBRA-2 reactor strip consisted of six reactors, operated at a working volume of 12 mL. The MBRA and MBRA-2 assembled systems were operated at ambient atmosphere at 37°C in a heated environmental chamber. The media were continuously replenished, and effluent was removed at a flowrate of 1.875 mL/hr.

### Pseudomonas aeruginosa culture in MBRA and MBRA-2.

P. aeruginosa strains PAO1 (MPAO1, University of Washington, Seattle) and PA14 (University of California, San Francisco) were inoculated from a streak plate into 5 mL Miller Luria broth (LB, Millipore Sigma) and incubated overnight at 37°C, shaking at 220 rpm. Cultures were diluted to an optical density at 600 nm (OD_600_) of 0.05. For the initial experiment testing P. aeruginosa biofilm growth in the MBRA, PAO1 was inoculated at 10^5^ CFU/mL in LB media. For comparison between growth in the MBRA and MBRA-2, PAO1 and PA14 were inoculated at 10^5^ CFU/mL in M9 minimal salts (BD, Difco) complemented with casamino acids (BD, Bacto, 5 g/L) and 1 mM tryptophan (MP Biomedicals).

### P. aeruginosa quantification from modified M9 media cultures.

A 1 mL sample was taken from each MBRA and MBRA-2 reactor well at 3, 6, 9, 12, 24, and 48 h postinoculation. Aggregates were mechanically disrupted by pipetting using wide-bore pipette tips (USA Scientific), followed by water bath sonication (Branson) for 10 min, and a secondary mechanical disruption using wide-bore pipette tips. Disrupted samples were serially diluted, spotted onto Pseudomonas isolation agar (PIA, BD Difco), incubated at 37°C overnight, and counted to determine CFU/mL.

### Statistical analysis of P. aeruginosa growth.

Statistical comparison of P. aeruginosa growth between MBRA and MBRA-2 was conducted in GraphPad Prism (version 9.4.1) using ANOVA with Šídák’s correction for multiple comparisons. For all analyses, *P* < 0.05 were considered statistically significant.
