# Supplementary material for: MBRA-2: a Modified Chemostat System to Culture Biofilms
Source: Microbiol Spectr. 2022 Dec 7;11(1):e02928-22. doi: 10.1128/spectrum.02928-22 (PMC9927502; doi:10.1128/spectrum.02928-22)
Supplement: Supplemental file 1 — Text S1. Download spectrum.02928-22-s0001.pdf, PDF file, 1.1 MB [file spectrum.02928-22-s0001.pdf]

Supplemental Material for

"MBRA-2: a modified chemostat system to culture biofilms"

Justin N. Jens[a], Daniel J. Breiner[a], Rachel L. Neve[b],

Matilda M. Fiebig[a], and Vanessa V. Phelan[a]

[a] Department of Pharmaceutical Sciences, Skaggs School of Pharmacy and Pharmaceutical Sciences, University of Colorado - Anschutz Medical Campus, Aurora, CO, 80045, USA

[b] Department of Immunology and Microbiology, School of Medicine, University of Colorado - Anschutz Medical Campus, Aurora, CO, 80045, USA

Materials to assemble an MBRA-2 (one reactor strip with two media rails):

Part #	Description	Manufacturer Number	Amount Required	Source	Notes
1	MBRA-2 lid		1	ProtoLabs	Threads for fittings must be manually introduced using a ¼”-28 hand tap tool
2	MBRA-2 reactor strip		1	ProtoLabs	
3	Custom silicone gasket		1		
4	Media rail		2	ProtoLabs	Threads for fittings must be manually introduced using a ¼”-28 hand tap tool
5	8/32 1” socket head cap screws		8	Hardware Store	Also called Allen bolt or hex socket cap screws
6	8/32 square nuts		8	Hardware Store	
7	Male nylon luer fitting with ¼”-28 threads	EW-45505-82	38	Cole-Parmer	Fittings for MBRA-2 lid, 2 6-port media rails, media/waste bottles
8	Female nylon luer fitting with 1/16” hose barb adapter	EW-45502-00	24	Cole-Parmer	Fitting to connect two-stop pump tubing to either a media rail or to C-flex tubing from reactor strip
9	Female nylon luer fitting with 1/8” hose barb adapter	EW-45502-04	12	Cole-Parmer	Fitting to connect reactor strip to C-flex tubing
10	Male nylon luer fitting with 1/8” hose barb adapter	EW-45505-04	12	Cole-Parmer	Fitting to connect C-flex tubing from reactor strip to pump tubing
11	Male PEEK dibafit adapter with ¼”-28 threads and 1/8” hose barb adapter	EW-21941-49	4	Cole-Parmer	Fittings for C-flex tubing to connect media rails to media or waste bottles
12	Tygon 2-stop pump tubing 1.14 mm ID	MFLX-96460-30	6	VWR	Effluent (red/red stops)
13	Tygon 2-stop pump tubing 0.89 mm ID	MFLX-96460-26	6	VWR	Inflow (orange/orange stops)

14	C-Flex tubing, clear, 1/8” ID x ¼” OD	MFLX06422-05	12: 20 cm length<br>2: variable length	VWR	12: reactor wells to media rails<br>1: media bottle to media rail<br>1: waste bottle to media rail
15	PTFE Diba Omnifit tubing, 1/8” OD x 1.5 mm ID	EW-21942-76	6: 25 mm length<br>6: 10 mm length<br>1: 45 cm length<br>1: 15 cm length	Cole-Parmer	25 mm: for effluent fittings<br>10 mm: for inflow fitting<br>45 cm: for media bottle<br>15 cm: for waste bottle
16	PTFE cylindrical stirring bar	Z328936	6	Millipore Sigma	
17	Sterile PTFE 0.22 µm syringe filter, 13 mm membrane diameter	50-202-071	8	Fisher	6 for reactor wells<br>1 for media bottle<br>1 for waste bottle
18	Diba Omnifit Q-series two female port GL45 threaded bottle cap	EW-21943-00	2	Cole-Parmer	1 for media bottle<br>1 for waste bottle
19	5 L glass laboratory bottle with GL45 threaded caps	FB-800-5000	2	Fisher	Any brand/size can be used, but GL45 threads required
20	24-channel peristaltic pump	EW-78000-41	2	Cole-Parmer	Any brand of low flow peristaltic pump can be used
21	2MAG MIXdrive 60 point stirrer	Z742334-1EA	1	Millipore Sigma	Required if stirring. Otherwise, any surface with similar dimensions will work.
22	MBRA holders		2	ProtoLabs	Ref. 1
23	¼”-28 hand tap tool		1	Hardware Store	For creating threads in reactor strip and media rails
24	9/64” hex wrench		1	Hardware Store	For turning screws to tighten nuts to attach lid to reactor strip
25	Instant mix epoxy		1	Hardware Store	For gluing tubing to inflow and effluent fittings
26	Spex VapLock male PTFE Port Plug ¼”-28	EW-12020-47		Cole-Parmer	Number needed to plug unused media rail ports

Notes:

1. These instructions for assembling the MBRA-2 are heavily derived from the instructions for assembling the MBRA provided in ref 1.

2. The MBRA-2 lid, reactor strip, holders, and media rails were manufactured by stereolithography using DMS Somos Watershed XC 11122 resin by ProtoLabs (www.protolabs.com). Any facility with similar capabilities can be used to fabricate these parts. All computer assisted design (CAD) files for the MBRA-2 lid, reactor strip, and media rails were deposited into the Zenodo repository (10.5281/zenodo.6909500). The CAD file for the MBRA and MBRA holders can be requested from the authors of ref 1.

3. The threads required for the fittings must be created on the MBRA-2 lid and media rails before assembly using a hand tap tool. The threads are not produced during production.

4. The die cast silicone gasket was cut in-house, but a variety of commercial vendors offer custom die cut gasket products. The file for the gasket design was deposited into the Zenodo repository (10.5281/zenodo.6909500).

5. Before use, visually assess all components for cracks, broken threads, and significant wear and tear. Do not use if there a cracks or broken threads that compromise the function of the MBRA-2 or media rails.

6. Spring loaded 4.5” bent nose or long nose jewelry pliers can be used to provide torque on the fittings to tighten them if accessing the fittings by hand is difficult due to limited space.

7. Wrapping the threads of the fittings with PTFE tape (plumber’s tape) reduces overtightening and breakage of fittings and increases airtightness of the system.

8. A video illustrating how to assemble the MBRA-2 with media rails accompanies this supplemental document (**Video S1**)

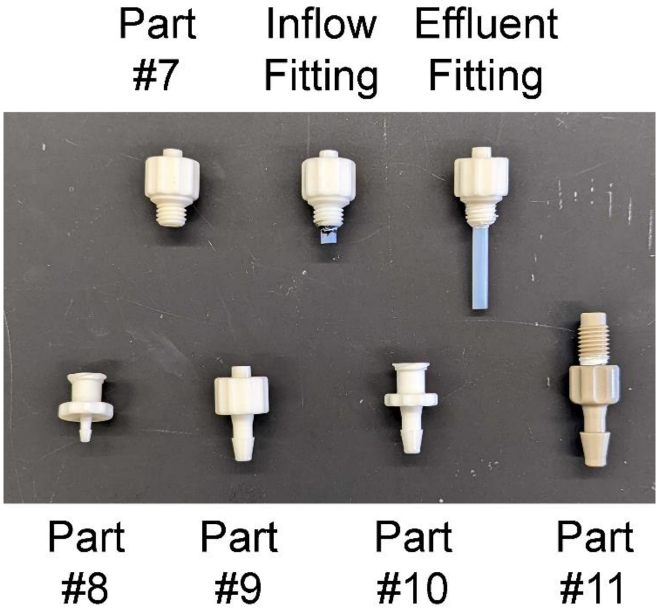

Figure S1. Part numbers for the different fittings used during assembly of the MBRA-2 and media rails.

Procedure for MBRA-2 assembly (1 reactor strip with 2 media rails):

1. **Prepare reactor strip inflow fittings (Figure S1, 6 fittings/reactor strip)**
    a. Securely insert a 10 mm long piece of PTFE tubing (Part #15) into a male luer fitting with ¼”-28 threads (Part #7, **Figure S1**).
        i. The small length of PTFE tubing of an inflow fitting forces the media into small droplets, which is required to prevent motile bacteria from climbing the inflow tubing.
    b. Using a pipette tip or toothpick, apply a thin layer of epoxy (Part #25) at the bottom edge of the threads of the luer fitting to secure the PTFE tubing in the fitting**.**
    c. Allow the epoxy to cure.

2. **Prepare reactor strip effluent fittings (Figure S1, 6 fittings/reactor strip)**
    a. Securely insert a 25 mm long piece of PTFE tubing (Part #15) into a male luer fitting with ¼”-28 threads (Part #7, **Figure S1**).
        i. The length of the PTFE tubing used in the outlet fitting determines the final working volume of the culture in the reactor well. The length of the tubing can be adjusted to alter the working volume of the reactor well.
    b. Using a pipette tip or toothpick, apply a thin layer of epoxy (Part #25) at the bottom edge of the threads of the luer fitting to secure the PTFE tubing in the fitting**.**
    c. Allow the epoxy to cure.

3. **Prepare C-flex tubing for the reactor strip (12 pieces of tubing/reactor strip)**
    a. Cut twelve pieces of C-flex tubing (Part #14) 20 cm in length
        i. To ensure that the length of tubing to and from all reactor wells is the same, the C-flex tubing can be cut into 6 pairs of 9, 11.5, 14, 16.5, 19, and 21.5 cm lengths. The tubing is then arranged from ascending length (from right to left) for inflow tubing and descending length (from right to left) for effluent tubing.
    b. Insert a female luer fitting with 1/8” hose barb adapter (Part #9, **Figure S1**) into one end of each piece of C-flex tubing.
    c. Insert a male luer fitting with 1/8” hose barb adapter (Part #10, **Figure S1**) into the other end of each piece of C-flex tubing.

4. **Prepare inflow 2-stop pump tubing (6 pieces of tubing/reactor strip)**
    a. Insert a female luer fitting with 1/16” hose barb adapter (Part #8, **Figure S1**) into each end of each 0.89 mm ID 2-stop tubing (Part #13, orange/orange color-coded stops).

5. **Prepare effluent 2-stop pump tubing (6 pieces of tubing/reactor strip)**
    a. Insert a female luer fitting with 1/16” hose barb adapter (Part #8, **Figure S1**) into each end of each 1.14 mm ID 2-stop tubing (Part #12, red/red color-coded stops).

6. **Assemble media rails (Figure S2)**
    a. NOTE: Assembly of six port media rails is described below. The same procedure is used for all media rail configurations.
    b. Screw male luer fittings with ¼”-28 threads (Part #7, **Figure S1**) into each of the ports on the long edges of the media rails (Part #4).
        i. If fewer ports are needed, PTFE Port Plugs (Part #26) can be used to stopper the unused ports of the media rail.
    c. Screw a male PEEK dibafit adapter with ¼”-28 threads and a 1/8” hose barb nylon (Part #11, **Figure S1**) into the port on the short edge of each media rail.
        i. Alternatively, screw a male luer fittings with ¼”-28 threads (Part #7, **Figure S1**) into the port on the short edge of each media rail (Part #4). Screw a female luer fitting with a 1/8” hose barb adapter (Part #9, **Figure S1**) onto the male luer fitting.

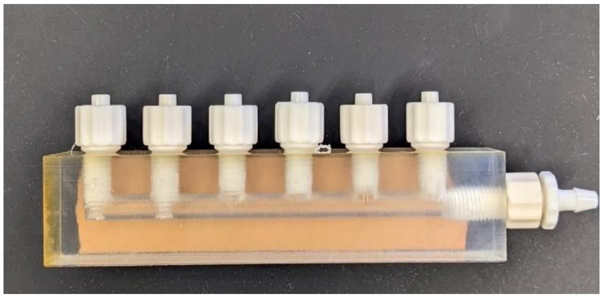

Figure S2. Assembled 6 port media rail.

7. **Assemble the MBRA-2 reactor strip (Figure S3)**
    a. If desired, place a stirrer (Part #16) in each reactor well
    b. Sandwich the silicone gasket (Part #3) between the MBRA-2 reactor well strip (Part #2) and lid (Part #1), aligning the screw holes.
    c. Tilt the MBRA-2 reactor strip so that it is resting at a 45° angle.
    d. Insert 8/32" square nuts (Part #6) into each of the four rectangular holes on the long side of the MBRA-2 reactor strip.
    e. Insert 8/32 1" socket head cap screws (Part #5) into each of the four screw holes on the top of the MBRA-2 reactor strip. Press down on the screws until they touch the square nuts.
    f. Using a 9/64" hex wrench (Part #24), turn the screws into the nut until tightened. Do not overtighten.
        i. The square nut should move up the screw as the screw is turned. If the screw does not catch the nut, the MBRA-2 reactor strip can be turned upside down or the orientation of the nut can be changed (sometimes the screw hole of the nut is off-center) to aid in assembly.
    g. Turn the MBRA-2 reactor strip 180° and repeat steps 7c-7f on the other side to fully secure the gasket between the lid and reactor strip.

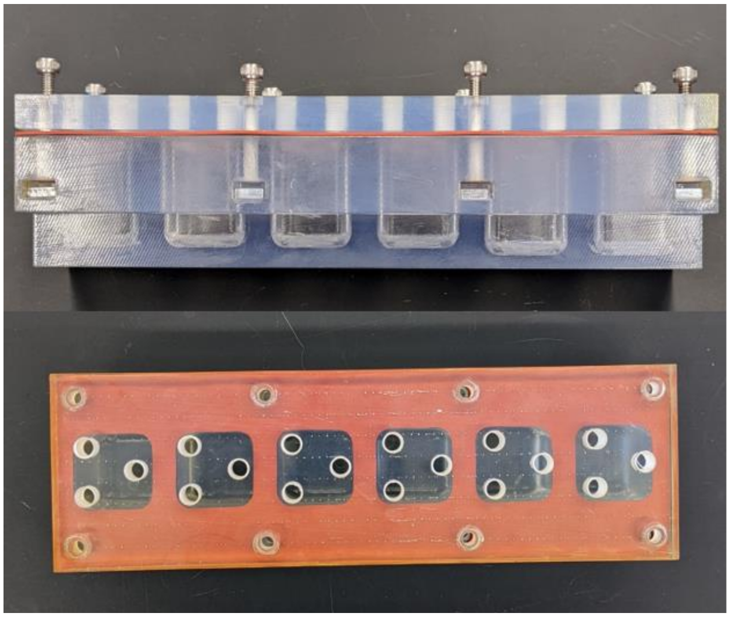

Figure S3. Lid, gasket, and reactor wells sandwiched together with screws and nuts inserted (top). Orientation of lid ports for screwing fittings into MBRA-2 reactor strip (bottom).

8. **Screw the inflow, effluent, and sampling fittings into the MBRA-2 reactor strip**
    a. Orient the MBRA-2 reactor strip so that each reactor well has two ports on the left and one port on the right.
    b. Screw reactor strip inflow fittings (from Step 1, **Figure S1**) into the top left port of each reactor well until finger tight.
    c. Screw reactor strip effluent fittings (from Step 2, **Figure S1**) into the right port of each reactor well until finger tight.
    d. Screw male luer fittings with ¼”-28 threads (Part #7, **Figure S1**) into each of the remaining ports.

9. **Attach C-flex and pump tubing to the inflow and effluent fittings of the MBRA-2 reactor strip** (**Figure S4**)
    a. Connect inflow tubing:
        i. Screw the female luer fitting of C-flex inflow tubing (from Step 3) into the male inflow fitting (Step 8b) of each reactor well until finger tight.
        ii. Screw the female luer fitting of 0.89 mm ID 2- stop tubing (from Step 4, orange/orange) into the male luer fitting of the C-flex inflow tubing of each reactor well until finger tight.
    b. Connect effluent tubing:
        i. Screw the female luer fitting of C-flex effluent tubing (from Step 3) into the male effluent fitting (Step 8c) of each reactor well until finger tight.
        ii. Screw the female luer fitting of 1.14 mm ID 2- stop tubing (from Step 5, red/red) into the male luer fitting of the C-flex effluent tubing of each reactor well until finger tight.

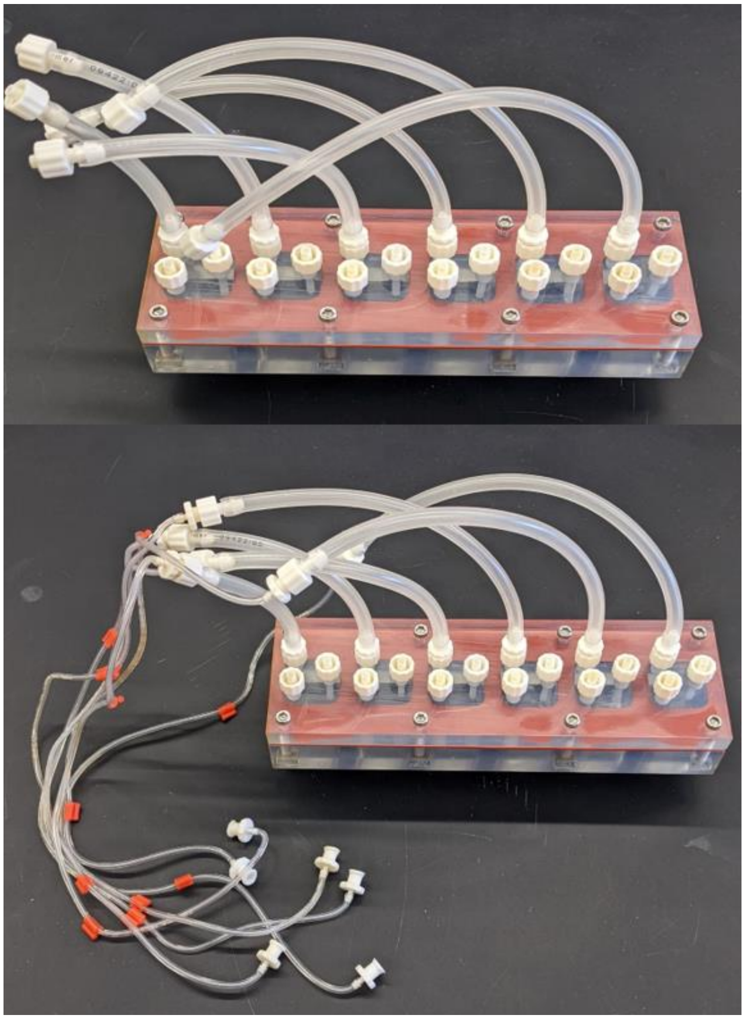

Figure S4. Inflow C-flex tubing connected to male inlet fittings on MBRA-2 reactor strip (top). 2-stop pump tubing attached to inlet C-flex media tubing (bottom). Effluent tubing is attached in an identical manner.

10. **Connect the MBRA-2 reactor strip tubing to media rails (Figure S5)**
    a. Inflow:
        i. Place a media rail (from Step 6) to the left of the assembled MBRA-2 reactor strip (from Step 9).
        ii. Screw the untethered female luer fittings of the 0.89 mm ID 2- stop tubing (orange/orange) from each reactor well into the male luer fittings of the media rail until finger tight.
    b. Effluent:
        i. Place a media rail (from Step 6) to the right of the assembled MBRA-2 reactor strip (from Step 9).
        ii. Screw the untethered female luer fittings of the 1.14 mm ID 2- stop tubing (red/red) from each reactor well into the male luer fittings of the media rail until finger tight.

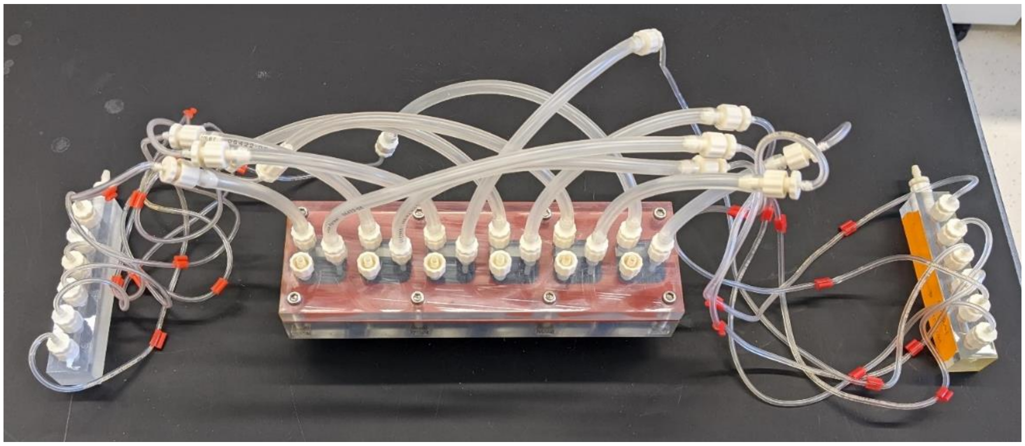

Figure S5. Assembled MBRA-2 reactor strip connected to two media rails (left: inflow, right: effluent).

11. **Assemble media and effluent bottle caps (Figure S6) and attach caps to bottles for sterilization**
    a. Screw in a male PEEK Dibafit adapter with ¼”-28 threads and 1/8” hose barb adapter (Part #11, **Figure S1**) into a port of a Diba Omnifit Q-series two female port GL45 threaded bottle cap (Part #18). Repeat for the second cap.
    b. Screw in male luer fittings with ¼”-28 threads (Part #7, **Figure S1**) into the second port of the bottle caps
    c. Media bottle cap:
        i. Fit a 45 cm length of Diba Omnifit PTFE tubing (Part #15) into the hole underneath the PEEK Dibafit adapter of one of the bottle caps.
            1. The length of the PTFE tubing needed for the media bottle cap is dependent upon the height of the bottle used for the medium. A 45 cm length of tubing is sufficient to reach the bottom of a 5 L bottle.
        ii. Screw the media source cap onto an empty, sterile 5L bottle (Part #19) for autoclaving.
            1. Alternatively, the cap and tubing can be wrapped securely in aluminum foil.
    d. Effluent bottle cap:
        i. Fit a 15 cm length of Diba Omnifit PTFE tubing (Part #15) into the hole underneath the PEEK Dibafit adapter of the second bottle cap.
            1. The length of the PTFE tubing for the effluent bottle can be any length.
        ii. Screw the effluent cap onto an empty, sterile 5L bottle (Part #19) for autoclaving and to serve as a collection vessel for the effluent.
            1. Alternatively, the cap and tubing can be wrapped securely in aluminum foil.

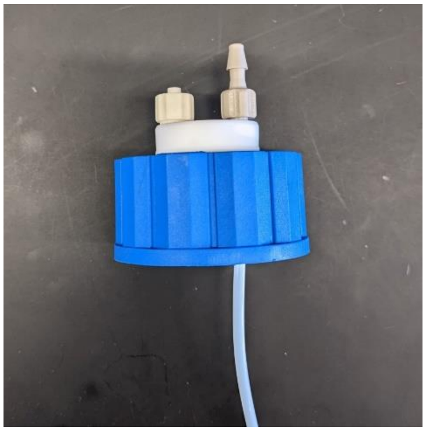

Figure S6. Assembled media/effluent cap.

12. **Connect MBRA-2 to media and effluent bottles through media rails**
    a. Inflow:
        i. Connect a length of C-flex tubing (Part #14) from the 1/8” hose barb adapter of the male PEEK dibafit adapter on the media bottle cap (from Step 11) to the 1/8” hose barb adapter of the male PEEK dibafit adapter of the inflow media rail (from Step 10)
            1. The length of C-flex tubing must be long enough to reach from the medium source bottle to the inflow media rail as measured from the final set-up of the MBRA-2 (Step 14).
    b. Effluent:
        i. Connect a length of C-flex tubing (Part #14) from the 1/8” hose barb adapter of the male PEEK dibafit adapter on the effluent bottle cap (from Step 11) to the 1/8” hose barb adapter of the male PEEK dibafit adapter of the effluent media rail (from Step 10)
            1. The length of C-flex tubing must be long enough to reach from the effluent media rail to the waste bottle as measured from the final set-up of the MBRA-2 (Step 14).

13. **Sterilize the fully assembled MBRA-2 with media rails and 5L bottles (Figure S7)**
    a. Cover all sampling ports on the MBRA-2 reactor strip and the male luer fittings on the media and effluent bottles with aluminum foil.
    b. Place the entire MBRA-2 assembly (bottles, reactor strip(s), tubing, and media rails) in a large autoclave bin.
    c. Cover the autoclave bin with aluminum foil.
    d. Autoclave the assembled MBRA-2 apparatus at 121ºC, ≥ 15 psi for 20 minutes, with slow exhaust.

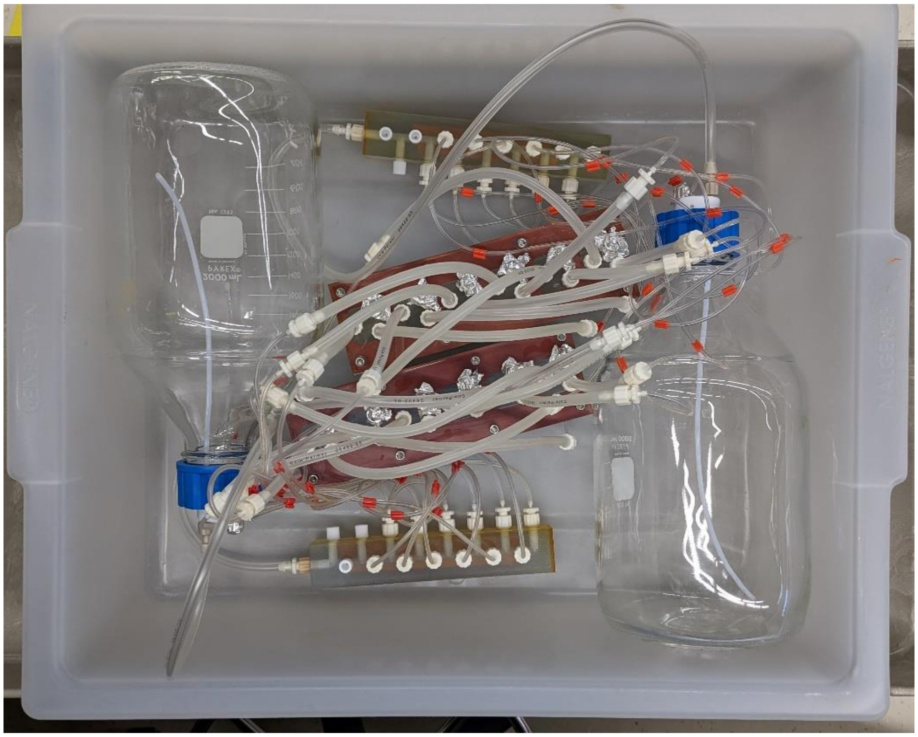

Figure S7. Assembled MBRA-2 apparatus (bottles, reactor strip, tubing, and media rails) in a large autoclave bin. The autoclave bin is covered with aluminum foil before sterilization.

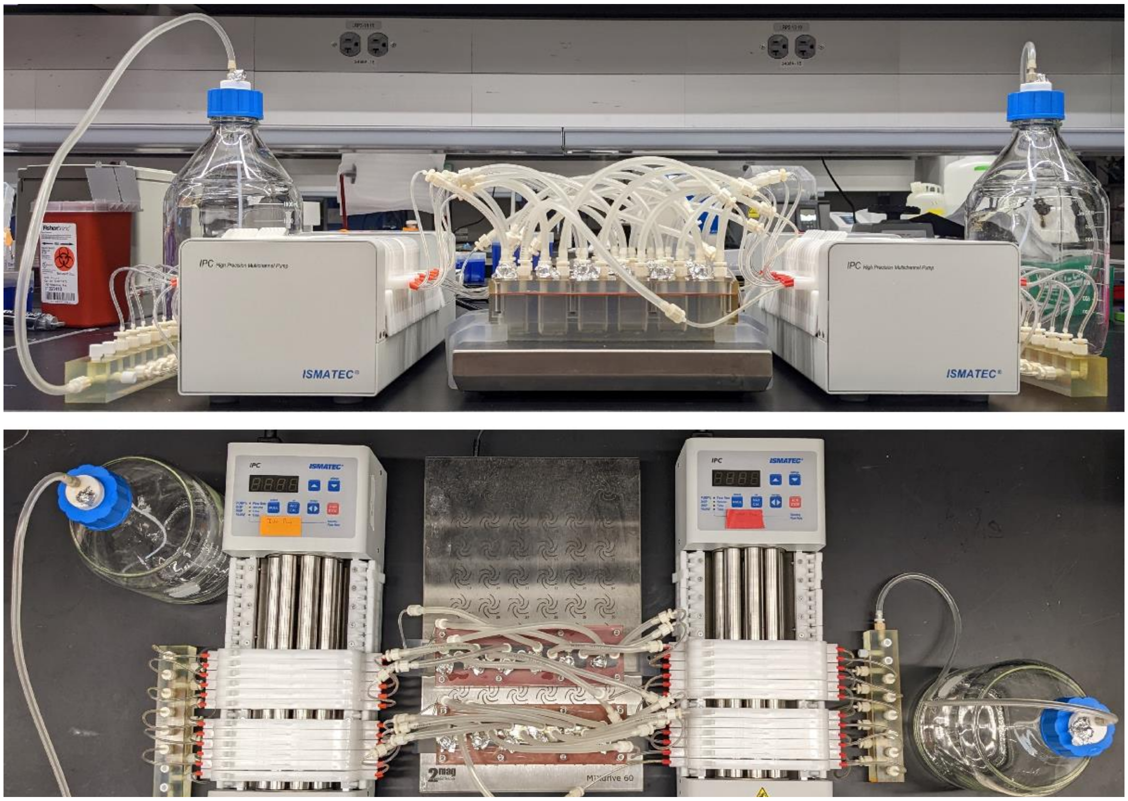

Figure S8. Assembled MBRA-2 with media rails set-up for use (top: profile view; bottom: top-down view) with flow from media bottle (left) to effluent bottle (right).

14. **Set-up the MBRA-2 apparatus for use (Figure S8)**
    a. Fit the MBRA-2 reactor strip into the MBRA holders (Part #22) situated on the 60-spot magnetic stir plate (Part #21), situating the media bottle and inflow media rail on the left inflow peristaltic pump (Part #20) and the effluent bottle and media rail on the right of the effluent peristaltic pump (Part #20).
        i. If using stir bars, ensure the reactor wells are aligned with the stirring positions.
    b. Tighten all fittings on the MBRA-2 reactor strip, media rails, and bottle caps.
    c. Fit the 2-stop inflow and effluent pump tubing into the clamps of the peristaltic pumps.
    d. Carefully transfer the media bottle cap (from Step 11) from the 5L sterile bottle to a 5L bottle containing sterile media.
    e. Remove the foil from the sampling ports and bottle caps and replace with 0.22 µm syringe filters (Part #17)

15. **Run the MBRA-2:**
    a. Verify that all connections are finger tight.
    b. Lock the inflow and effluent pump tubing clamps onto the peristaltic pumps.
    c. Turn on the source pump, set the flow to the maximum rate, and allow the reactor wells to fill until 90% full.
    d. Turn on the effluent pump, set the flow to the maximum rate, and allow the reactor well volume to equilibrate at the bottom of the outlet fitting tube.
    e. Reduce the source and effluent pump flow to 1.875 mL/hr and 3.75 mL/hr, respectively.
        i. The effluent flow rate should be twice the rate of the inflow rate.
    f. Check for bubbles and leaks and adjust fittings as needed.
    g. Turn off the pumps and leave the reactors overnight to ensure that the system is sterile.
    h. Prepare inoculum as desired.
    i. Turn on the pumps and start flow.
    j. Loosen the 0.22 µm syringe filters of the sampling ports and inoculate each reactor well via the sampling port. Sample as desired through the sampling port.

16. **Cleaning the MBRA-2:**
    a. Replace the media bottle with a bottle containing 20% bleach.
    b. Set the source and effluent pump flow to 1mL/min and 2 mL/min, respectively.
    c. Allow the reactors to fill with 20% bleach.
    d. Flow the bleach through the tubing and reactors for 60 minutes
        i. If cleaning overnight, return flow rates to 1.875 mL/hr and 3.75 mL/hr
    e. If desired, replace the 20% bleach with water and repeat steps 14a - 14d.
    f. Disassemble the MBRA-2 and media rails.
        i. Unscrew all tubing connections, remove all fittings, and dispose of used tubing in proper waste streams.
    g. Empty the MBRA-2 reactor wells using the proper waste stream.
        i. Dislodge residual biofilm from the reactor wells using a soft bristled brush.
        ii. Residual biofilm in the inflow and effluent PTFE tubing can be removed with a pipe cleaner.
    h. Rinse the MBRA-2 lid, reactor wells, gasket, media rails, and fittings with water. Allow to dry before storage.
        i. The MBRA-2 reactor strip, gasket, media rails, screws, nuts, and fittings (including those with PTFE tubing attached) can be reused until there are visible signs of wear that affect performance.
            1. Screws and nuts should not be bleached as they will rust.

REFERENCES

1. Auchtung JM, Robinson CD, Farrell K, Britton RA. 2016. MiniBioReactor Arrays (MBRAs) as a Tool for Studying C. difficile Physiology in the Presence of a Complex Community. Methods Mol Biol 1476:235-58.
